# Suitability of incorporating plantain stem cellulose nanocrystals into cmc/gelatin film for packaging applications

**DOI:** 10.1038/s41598-025-18796-z

**Published:** 2025-09-29

**Authors:** Emmanuel Chukwudi Nwanna, Paul Chukwuka Eze, Louis Chukwuemeka Orakwe, Emmanuel Chibundo Chukwuma, Chike Pius Nwachukwu, John Ikedinachukwu Maduegbuna

**Affiliations:** 1 Department of Agricultural and Bioresources Engineering, Nnamdi Azikiwe University, Awka, Nigeria; 2 Department of Agricultural and Bioresources Engineering, Enugu State University, Enugu State, Nigeria; 3 Agriculture department, Faculty of Agriculture, Environmental Management, and Renewable Energy, University of Technology and Arts, Byumba, Rwanda; 4 Future Africa Institute, University of Pretoria, Pretoria, South Africa

**Keywords:** Plantain stem, Cellulose nanocrystals, Gelatin, Carboxymethyl cellulose, Nanocomposite, Engineering, Environmental sciences, Materials science, Nanoscience and technology

## Abstract

Plastic waste littering from food package poses severe pollution on the streets of most countries. Research on the high-value application of plantain stems, an abundant, easily available, and renewable agricultural waste for alternative bio-packaging is urgent and imperative. The present study investigated the application of natural waste products with outcomes of cellulose nanocrystals (CNC) content on various physical properties of CNC, Carboxymethyl cellulose (CMC), gelatin barrier layers, including transmission electron microscopy (TEM), Fourier transform infrared spectroscopy (FTIR), water absorption, x-ray diffraction (XRD), scanning electron microscopy (SEM) moisture uptake and total dissolved solid (TDS) as well as mechanical properties like thickness (THS), tensile strength (TNS) and elongation at break (EAB). The solution casting approach was effective in producing gelatin/CMC nanocomposites reinforced with CNC. According to the study’s findings, the CMC/gelatin and CNC films were needle-shaped, with lengths ranging from 81 to 286 nanometers, cross-sections from 8 to 21 nanometers, a dimensional proportion of 17, and a degree of crystallinity of 0.82 when observed under scanning electron microscopy. When 5 and 10 weight per cent of CNC were added, the CNC was proportionally dispensed throughout the network to produce equal barrier layers, showing that CNC and CMC/gelatin were well matched. The thickness (THS) of the nanocomposite films grew from 0.1 μm to 0.11 μm, and their tensile strength (TNS) also grew from 4.27 MPa to 7.22 MPa with the supplement of CNC. Additionally, their elongation at break (EAB) dropped as well, falling from 94.36 to 57.21%. The nanocomposite films TDS dropped from 70 to 63% as well. The outcomes show that using gelatin/CMC reinforced with CNC has several benefits because it is a naturally occurring, affordable, and plentiful material that can replace a lot of products with petroleum and non-degradable bases.

## Introduction

Plantain stem is one of the major byproducts of plantain plantation; it currently lacks economic value apart from the use as natural manure in farms in most developing countries of the world. It is seen as a major waste after the plantain fruit has been harvested. Because each plant only bears fruit once, plantain stems are waste biomass that is produced in vast quantities once the fruit is harvested. Plantain stem can therefore be used to create a range of valuable products, making it both highly available and an esteemed bio-material, yet underutilized source of cellulose. Several researchers in developing countries are advocating for the utilization of waste for circular economy and waste minimization in production processes^1,2^. Studies have shown that plastic material, a non-degradable packaging material is predominantly used in most developed and developing countries, this is a major contributor to environmental, socio-economic, and health challenge^3,4^. To minimize the impact of plastic pollution, various studies have considered measures to reduce the volume of plastic through various transformation studies^3,5^. Alternatives to plastics, rather than minimization strategies must be the focus of scientists in developing countries, with emphasis on naturally abundant waste material within a given locality^6^.

Scientists are searching for safe biodegradable alternatives to synthetic polymers derived from petroleum, because the current plastic materials, frequently employed in food packaging are of a major concern on food safety and environmental harm^1,7^. Edible and biodegradable films have the potential to be sustainable substitutes for non-biodegradable plastic packaging materials in the food industry because of their many advantages in reducing oxygen permeability, moisture loss, water movement, moisture absorption in the food matrix, and scent loss^8^. Lately, a lot of work has gone into creating edible and biodegradable films using natural biopolymers^9^. Because of their renewable qualities, non-toxicity, ease of accessibility, and biodegradability, natural polymers like nucleic acids^10^, proteins^11^, and modified polysaccharides like starch-based bioplastics^12^ are considered viable resources for the production of biodegradable packaging films. Because they are considered inexpensive; polysaccharides might be the most promising biopolymer option. CMC is one of the most widely used components in the creation of bioplastics. This synthetic polymer is non-toxic, transparent, and has excellent biodegradability and biocompatibility. Because of its gelling, thickening, stabilizing, bulking, and emulsifying qualities, CMC is one of the cellulose ether compounds that are most frequently employed in culinary applications^13^. A sodium carboxymethyl group (CH2COONa) is added to the cellulose molecule to make it water-soluble. The primary factors influencing the widespread use of CMC are its non-toxicity, flocculating ability, transparency, low cost, and viscosity^14^. Because of its polymer structure and high molecular weight, CMC can also be used to create ideal biodegradable films^15^. However, gelatin is a soluble, edible substance and renewable, and is also utilized to create biodegradable films. It is an animal-based protein that is widely utilized in the pharmaceutical and culinary industries. It is manufactured from collagen using alkaline or acid hydrolysis^16^. Accessibility, flexibility, gas flow barrier, optical qualities, functional attributes, and price effect are only a few of the unique factors that determine the use of gelatin in packaging^17^. The production of bio-packing food material against the synthetic products will have enormous benefits in environmental sustainability, human health, and social life-style.

CNC is one of the most researched bio-based fillers; they are cellulose components with at least one dimension less than 100 nm^18^. To produce the CNC, enzymes or acids hydrolyze cellulose fibers. They are used as reinforcements in polymer nanocomposites because of their unique properties, which include huge surface area, biodegradability, high modulus, environmental benefits, and the ability to form a highly porous structure^19,20^. One can separate CNCs from a variety of natural cellulose sources. However, plant biomass is the most readily available and abundant of these sources. As a result, they present the best chance for CNC production on a big scale. However, it should be remembered that in addition to the separation techniques employed, the source from which the nanocellulose was isolated affects its shape, size, and degree of polymerization^18^. The plant’s inedible components, which include pseudo-stems and leaves, account for over 88% of its total weight^21^, are thrown away as trash. Plantain stem residues amount to approximately 29.0 million tons annually in China alone^22^. The cellulose fiber content of the plantain stem is high^23^. However, despite being investigated for the creation of environmentally acceptable packaging materials, bioplastics have many shortcomings, including low mechanical and barrier characteristics. However, by combining biopolymers with two or more polymers, these qualities can be enhanced. It’s among the best ways to make new biomaterials with the appropriate characteristics. Biopolymer-blended films typically have altered characteristics^24^. Thus, since blending improves the qualities of biodegradable polymers for packaging, continuous development is necessary, particularly in attributes like mechanical properties, biodegradation, and water vapour permeability. Additionally, some research work has been done to investigate how crosslinking could improve the material properties of biodegradable films. Crosslinking, either chemical or physical, was used to accomplish this^25,26^. Polymers need to be blended with other substances, usually fillers, to modify their characteristics so they can better meet the demands of different packaging applications. Additionally, biodegradable polymers’ mechanical qualities are improved and their hydrophilic nature is diminished by filler reinforcement^27^. This study aligned with previous studies, to investigate methods to enhance the mechanical properties of bio-packaging material, this is critical in durability of the bio-packaging product.

Researchers have been paying close attention to the use of bio-based and nano-sized fillers lately. The literature is not overly abundant when it comes to the application of CNC to improve the properties of popular film-forming mixes such as CMC-gelatin. Thus, the premise of this work is that a nuisance plant (plantain stem) can be made more valuable for food packaging. The research’s anticipated output will provide interesting applications for plantain stems as well as help address pressing environmental issues relating to petroleum-based, non-biodegradable plastic films used in food packaging.

## Materials and methods

The components were sourced from the Anambra State Chemical Market in Onitsha, Nigeria. Glycerol (98%), benzene (99%), acetic acid (98%), ethanol (99%), carboxymethyl cellulose (molecular weight: 115,000 g/mol; degree of polymerization: 1700–18,000), sodium hydroxide, calcium chloride (96%), sodium chlorite, potassium sulphate (99%) and sulphuric acid (99%) are in the analytical grades. The plantain stem fiber came from Okeanyanwu’s Plantation Farm in Awka Town, Awka South Local Government Area, Anambra State, Nigeria.

### Extraction of cellulose from plantain stem

Plantain stems were gotten from the plantation farm owned by Okeanyanwu in Awka town, Awka-South L.G.A., Anambra State. The fibers were dried, crushed, and sieved following an hour at 60 °C in distilled water with mechanical agitation. The fibers were prewashed and then soaked in a 4wt% sodium hydroxide solution with a fiber-to-liquor ratio of 1:20 and mechanical stirring for two hours at 80 °C. The fibers were then extensively washed with distilled water and dried to constant weight in an oven set at 100 °C. A solution containing 65 milliliters of water, 0.5 milliliters of acetic acid, and 0.6 g of sodium chlorite was applied to 1 gram of fiber after it had dried for four hours at 80 °C. Until the fiber turned white, a solution of sodium chlorite, acetic acid, and water was applied constantly every hour. After giving the fiber a thorough wash in distilled water, it was dried. A slightly modified version of this approach was used by previous researchers to extract the cellulose^28^.

### Cellulose nanocrystal (CNC) Preparation

A controlled hydrolysis using sulphuric acid was used to produce the CNC. With vigorous mechanical stirring and 45 °C for 30 min, dried cellulose was combined with a 64-weight per cent sulphuric acid (H_2_SO4) solution at a ratio of 1 g cellulose to 8.75 ml acid^29^. To stop and dilute the reaction, deionized water was used ten times the solution (10%). After centrifuging the cold suspension, the supernatant was extracted. After diluting the solid Material once more, the supernatant was centrifuged until it turned turbid. For a week, the turbid supernatant was dialyzed against deionized water until a pH of approximately 6.5-7 was achieved.

### Film Preparation

**Fig. 1:**
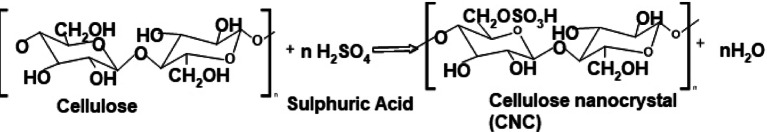
CNC synthesis.

**Fig. 2:**
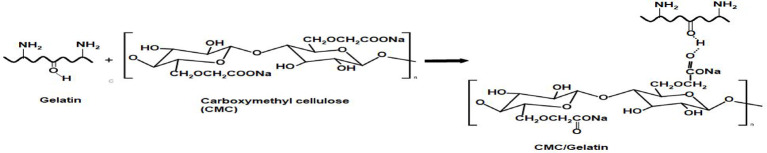
CMC and gelatin blending.

**Fig. 3:**
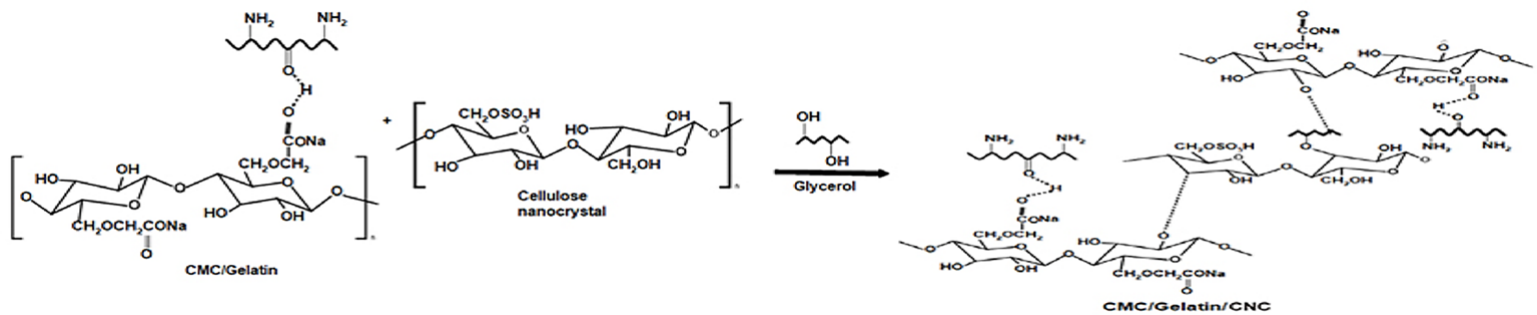
Development of the film.

The film was prepared via solution casting, a method akin to that employed by Mohammadi et al. (2019)^30^. In distilled water at 90 °C for 30 min, a preset weight of carboxymethyl cellulose (as listed in Table 1) was dissolved with continuous stirring until full dissolution was achieved. For 30 min, distilled water was used to dissolve a known weight of gelatin (as shown in Table 1) at 80 °C. At 90 °C, gelatin and carboxymethyl cellulose were mixed and vigorously stirred for 30 min. The required cellulose nanocrystals were added to room temperature glycerol, then drops of this mixture were added to the gelatin/carboxymethyl cellulose solution and was heated to 90 °C while being agitated. To allow the solvent to gradually evaporate, the resultant solution was then placed in a petri dish and left to air dry for 48 h at 25 °C. The water was completely evaporated from the films, and they were kept fresh in a desiccator until examination. Figures 1, 2 and 3 shows the CNC synthesis, CMC and gelatin blending and the development of the film.

### Using an experimental design with a design expert

The effect of gelatin, CNCs, and CMC concentration on the dependent variable (thickness, elongation at break, and tensile strength) was examined using a response surface approach with a central composite design. The dependent variables were then optimized once the level of the independent variables was determined using a three-factor, two-level experimental design. The CNC reinforcement was measured in relation to the total solid content of the film-forming solution (the combined amount of gelatin and CMC), while the concentrations of gelatin and CMC were measured at about 100 g of the film-forming solution. Previous studies^16,31–33^ indicate that the quantitative composition of the formulation was developed based on the expected influence of each component on the overall performance of the produced nanocomposite films. The CCD generated twenty trial runs with an alpha value of 1.76. Six center points, six axial points, and eight combinatorial points Make up these formulations. Design Expert Software version 10 was used to analyze the data and the tests were carried out at random.

### X-ray diffraction analysis of plantain stem

The cellulose nanocrystals were examined using a Siemens D-500 diffractometer exposed to 40 kV X-ray radiation. Scattering radiation was detected in the angular range (2ϴ) between 5° and 70° with a step size of 0.02 and a 30-second gap between each step. The working voltage was 40 kV, and the current was 40 mA, based on a technique described in the literature^31^. To determine the crystallinity percentage of the CNC, this data was displayed as a curve [35].

1 $$\:\mathbf{\%}\:\text{C}\text{r}\text{y}\text{s}\text{t}\text{a}\text{l}\text{l}\text{i}\text{n}\text{i}\text{t}\text{y}\:\text{I}\text{n}\text{d}\text{e}\text{x}=\frac{{P}_{AB}-{P}_{CD}}{{P}_{AB}}\times\:100\:$$

Where,

P_CD_ is the magnitude scattered by the sample’s amorphous portion and P_AB_ is the peak magnitude of the crystallographic plane.

### Transmission electron microscopy of plantain stem

A JEM-2100 transmission electron microscope operating at 80 kilovolts was used to study a drop of diluted CNC suspension that had been placed on a copper grid and let to dry. Using picture J 1.52 visualization software, the morphological picture of the CNC sample was analyzed for particle size by calculating and utilizing averages of the sizes of more than 100 CNC nanoparticles.

### Nanocomposite film morphological structural analysis

The dispersion of the CNC fillers within the polymer Matrix was examined using an SEM operating at 5 kilovolts on a VEGA 3 TESCAN. The film samples were vacuum-dried at 40 °C for a whole night before being cryogenically broken to reveal their cross-section for analysis. To improve contrast in the SEM, the samples were coated with a 5-nm layer of platinum before analysis.

### Chemical structural examination of the nanocomposite films

The chemical structure of the films was investigated using an Equinox 55 FTIR spectrometer equipped with a golden gate single attenuated total reflection (ATR) cell. The transmittance mode was used to capture the FTIR spectra, which covered the range of 4000–500 cm^−1^ at a resolution of 4 cm^−1^.

### Tensile examination

By ASTM Standard D882-09 (2009), tensile testing (Eq. 2) was performed using an M500-25CT Universal mechanical testing equipment. The samples were kept in a desiccator with calcium chloride desiccant for a full day before testing. Rectangular films of 100 mm by 20 mm were used, and the sample’s thickness was measured by averaging 10 randomly chosen locations on it using a portable micrometer with a precision of 0.01 mm. The films measure 0.21 ± 0.03 mm in thickness. Tensile strength was measured at 50 mm/min crosshead speed, using a 60 mm grip separation and 5 N pretension. The results for elongation at break were also calculated using (Eq. 3) using the same methodology.

2 $$\:\text{T}\text{e}\text{n}\text{s}\text{i}\text{l}\text{e}\:\text{s}\text{t}\text{r}\text{e}\text{n}\text{g}\text{t}\text{h}\:\left(\text{M}\text{P}\text{a}\right)=\frac{Load\left(N\right)}{Thickness\left(mm\right)\times\:Width\left(mm\right)}$$

3 $$\:\text{E}\text{l}\text{o}\text{n}\text{g}\text{a}\text{t}\text{i}\text{o}\text{n}\:\text{a}\text{t}\:\text{b}\text{r}\text{e}\text{a}\text{k}\:\left(\text{\%}\right)=\frac{Displacement\:at\:break}{Guage\:length}\times\:100$$

### Total dissolved solids

The sample (30 × 20 mm) was weighed before being immersed in 100 ml of room temperature water for a whole day in order to perform the film stability list. Following their removal, the samples were dried using a lint-free cloth and then put in a desiccator to finish drying. The sample was then weighed, and the dissolved solid was ascertained using gravimetry.

4 $$\;Percentage{\text{ }}weight{\text{ }}loss{\text{ }}\left( \% \right) = \;\frac{{{{\text{W}}_{\text{a}}} - {{\text{W}}_{\text{b}}}}}{{{{\text{W}}_{\text{b}}}}}{{\; \times 100\% }}$$

Where,

W_a_ = the film’s initial constant weight.

W_b_ = Final Constant Film’s Weight.

### Film’s water absorption

With a small adjustment, the film water absorption was tested in accordance with^31^. The film (30 × 20 mm) was weighed before it was placed in 100 milliliters of water. It was then taken out and weighed once more after 5, 10, 15, 20, 25, 30, 35, 40, 45, and 50 min.

5 $$Percentage{\text{ }}weight{\text{ }}loss = \;\frac{{{{\text{Z}}_2} - {Z_1}}}{{{Z_1}}}{{ \times 100\% }}$$

Where,

Z_1_ = Constant mass of the film at the beginning.

Z_2_ = Constant mass of the film at the end.

### Moisture uptake of the film

As stated by^31^, the moisture uptake was assessed using the [35] method. First, 20 millimeters by 20 millimeters dried films were primed for 24 h at zero per cent relative humidity with calcium sulphate. The films were weighed and then primed at 25 °C in a desiccator filled with a waterlogged solution of potassium sulphate to Maintain a 98% relative humidity. After that, the films were weighed repeatedly till they attained a stable weight at predetermined intervals. Equation 6 was then used to compute the moisture uptake.

6 $$Moisture{\text{ }}uptake{\text{ }}\left( \% \right){\text{ }} = \frac{{{Y_{1 - {Y_2}\;\;}}}}{{{Y_1}}}\; \times {\text{ }}100\%$$

Where,

Y_1_ = the sample’s weight after exposure to 98% relative humidity for a period of time.

Y_2_ = initial constant weight of the film.

### Oxygen transmission rate (OTR)

In accordance with ASTM D3985, the OX-TRAN Model 1/50 (MOCON, Minneapolis, MN, USA) was used for assessing the film’s OTR. The film specimens were put across two separate spaces to form a closed shield after being cured. Nitrogen (carrying gas) was used to cleanse one of the spaces, whereas oxygen (test gas) was used in the second. After entering the nitrogen flow via the film, oxygen was carried to a coulometric sensor. The total quantity of oxygen flowing across the film over a period of time interval was determined by the sensor. At 23 °C and 0% relative humidity (RH), the OTR results were reported in cubic centimeters per square meter per day (cc/m^2^/day).

## Result and discussion

### Diffraction pattern analysis

**Fig. 4:**
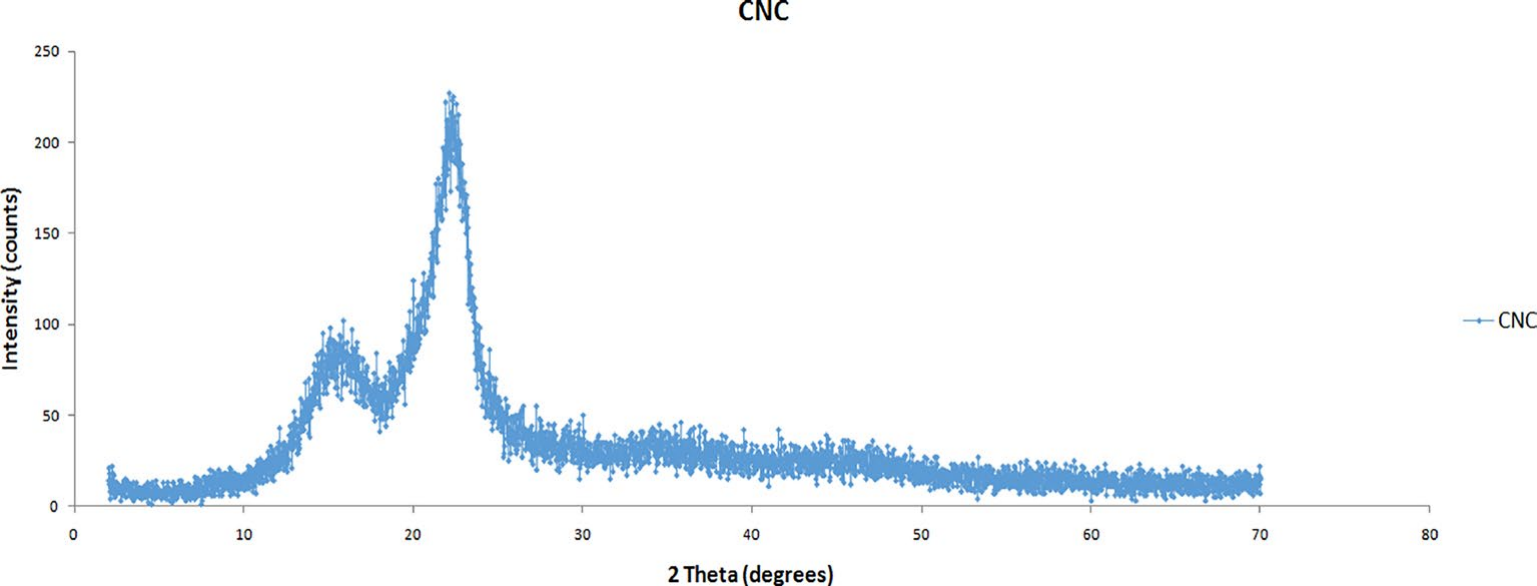
Diffraction micrograph of plantain stems CNC.

The CNC crystallinity produced from the plantain stem fiber was measured with XRD technque. The degree of crystallinity in natural fibers can be enhanced by employing acid to hydrolyze the non-crystalline components of them. As a result, after bleaching the plantain stem fiber, only the disordered cellulose was taken away. The amorphous zones are pierced by the hydronium ions, which promote the degradation of glycosidic linkages and release the discrete crystals, while the crystalline domains are more impervious to chemical corrosion (Alonzo et al., 2014)^35^. Crystallinity is a primary element influencing the scattering spectra of cellulose. Figure 4 displays the diffractogram of plantain stem fiber-derived cellulose nanocrystals. The crystalline structure of cellulose is related to the primary intensity peak found at the 2θ value of around 22.7^o^ in the x-ray diffraction spectrum of cellulose nanocrystals. Furthermore, cellulose’s crystalline structure is Linked to the lower peaks seen at 41^o^, which are likewise traits of cellulose^28^. Furthermore, observations have shown that the peaks located at 16.5^o^ have cellulose-specific properties^36^. Using Segal Eq. 1, the CNC crystallization index Made out of plantain stem fiber was established to be 82%. This is less compared to findings from potato peel (85%) by Chen et al. (2012)^27^, but it is greater compared to the results achieved by Wang et al. (2018)^38^ from Pueraria root residue (60%), from water hyacinth (72%)^31^ as well as the outcomes achieved by El-Miri et al. (2015)^28^ out of cane sugar bagasse (65%). The conditions under which the CNC was extracted during hydrolysis may also have contributed to the variation in the crystallinity index^36^.

### TEM examination

**Fig. 5:**
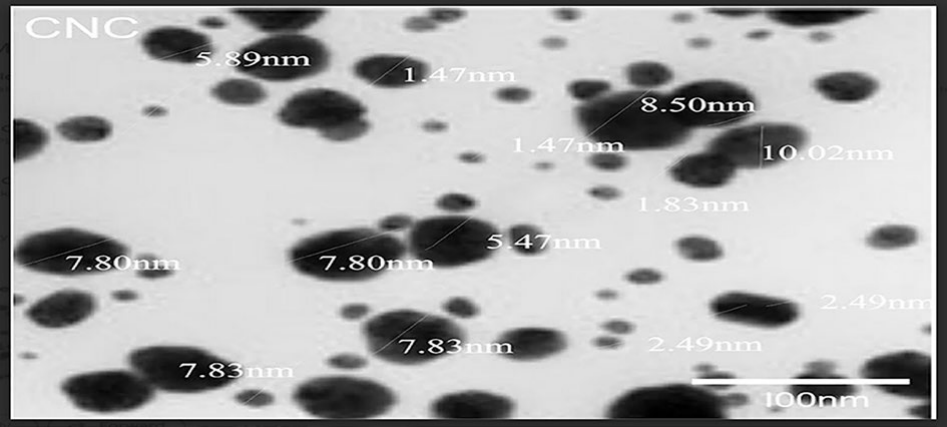
Transmission electron micrograph of the CNC.

Figure 5 presents a TEM examination micrograph from the CNC that was sourced from the bleached plantain stem strand, which helps to explain the dimensions as well as bodily makeup from the cellulose nanocrystals. The electron beam image demonstrates that the existence of nanometric cellulose particles with a globular form allowed for the extraction of cellulose nanocrystals from the plantain stem fiber. The simultaneous layering of the whisker-like fashioned fragments was caused by the CNC’s great aggregation capacity. Furthermore, it is evident that the CNCs in Fig. 5 overlap, which causes a significant amount of aggregation to form in the aqueous suspensions^31^. Additionally, it has been noted that the robust hydrogen interactions among the nanoparticles along with the drying stage during specimen preparation for TEM testing may cause the aggregated particles [19, 39]. It was discovered that the fractionated CNCs had an overall dimension of 31 nanometers alongside ranged in diameter from 10 to 20 nm. This is comparable to the range found in the CNC records (10–60 nm) out of Brasiliensis Imperata grass from the cotton stem (10 to 50 nanometers)^39,40^. The use of disparate extraction procedures and analytical processes makes data comparison challenging; furthermore, the dark area that is apparent in the TEM image possibly may be the consequence of non-cellulosic material around the plantain stem fiber, or it could have been pollutants added during testing or preparation.

### Scanning electron microscope examination

**Fig. 6:**
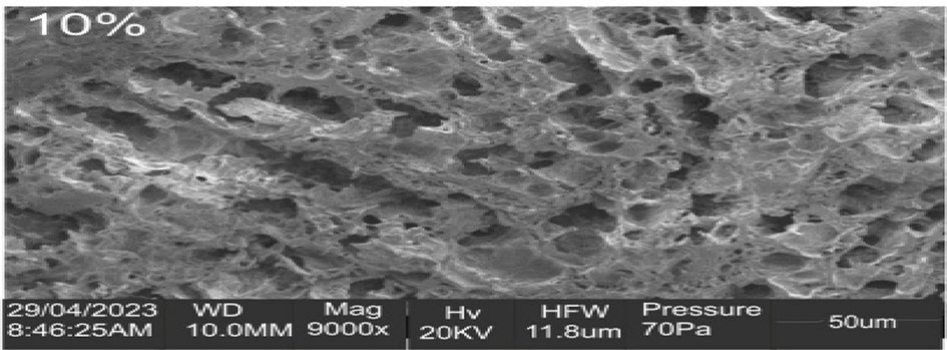
Scanning electron micrograph of the nano-scale films.

Figure 6 also displays an SEM picture demonstrating the uniformity of CNC particles throughout the gelatin-CMC blend. Inadequate homogeneity during film preparation may have led to some loose granules and discontinuous cracks in the micrograph of the unreinforced film (Fig. 6a)^42^. The film reinforced with 5 weight per cent CNC (Fig. 6b) showed better cohesive structure and interaction between CNC and Matrix, while the gelatin-CMC film reinforced with 10 weight per cent CNC (Fig. 6c) showed certain irregularities and a pronounced fracture formation. This might be the consequence of increased CNC intensification increasing agglomeration and decreasing dispersibility.

### Chemical makeup of the CNC

**Fig. 7:**
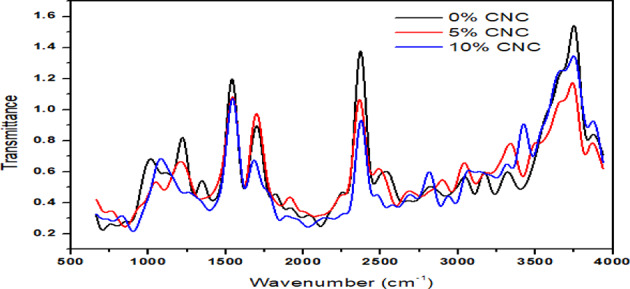
Plantain stems Fourier Transform Infrared Spectra of 0%, 5% and 10% CNC.

Figure 6 also displays an SEM picture demonstrating the uniformity of FTIR analysis of the nanocomposite film made of plantain stem fiber revealing the chemical structural makeup and changes of samples eliminated by various chemical techniques as shown in Fig. 7. The spectra’s peak at 849 cm − 1 is ascribed to the β-glycosidic bonds of the cellulose’s glucose chain^43^. This peak’s intensity suggests that several extracting procedures raised the cellulose content. The results found are consistent with previous research^38^. The C-O-C glycoside ether bond and the chain C-C stretching vibrations of the β−1,4-glycosidic chain connections that connect the units of D-glucose in cellulose are responsible for the peaks seen at 1160 cm − 1^43^. The peak at 1400 cm-1 originates from lignin’s hydroxyl group alongside C-H bending. The absorption peak between 2100 and 2400 cm-1 was brought on by the N-H bond and free O-H groups. The unbalanced vibration of stretching caused by the C-H bond is responsible for the vibrations found in the 2850 and 2930 cm − 1 region^31^. The hydrophilic characteristic of the fiber is indicated by the peak in the region 3916 and 3945 cm − 1, which corresponds to the O–H vibrations associated with the stretching of hydroxyl groups that are present in cellulose molecules. The results show that after the acid treatment, the crystalline region remained unaltered while the non-crystalline zone was eliminated, and the frequency readings are in excellent conformity with previous findings^38^.

### Mechanical attributes of the nanocomposite sheet

#### Tensile strength (TNS) of the nanocomposite sheet

For an improved tensile property, there must be a favorable surface connection between the CNC alongside the polymeric network with consistently mixed and uniformly distributed CNC. In contrast, run 16 (0-weight per cent film with 4.27 MPa), Run 2 (5-weight per cent CNC reinforced film with 7.22 MPa), alongside Run 14 (10-weight per cent CNC reinforced film with 7.19 MPa) observations in Table 1 unveil that CNC increased the films tensile strength. The positive contribution of CNC to the tensile strength was observed to diminish slightly when the CNC was raised from 5-weight per cent to 10-weight per cent. This could be due to inadequate dispersion, which is brought on by consolidated fragments that complementarily produce pressure spots in the network. Additionally, for runs 2 with 4 g of CMC and 7 g of gelatin having 7.22 MPa tensile strength and runs 10 with 8.19 g of gelatin and 2.41 g of CMC having 6.84 MPa tensile strength, which was closely evaluated, the significance of CMC to the tensile strength of the nanocomposite was shown to be greater compared to gelatin. According to previous researchers, the tensile strength of gelatin/CNC in plasticized films Made of Maize starch varied from 10.91 to 49.09 MPa [44].

**Table 1:** Experimental setup with actual alongside predicted outcomes.

Run	A: GEL. (g/100 g)	B: CMC (g/100 g)	C: CNC (%)	TENSILE STRENGTH (TNS)			ELONGATION AT BREAK (E_AB_)			THICKNESS (THS)		
				ACTUAL VALUE (MPa)	PRED. VALUE (MPa)	RESIDUAL	ACTUAL VALUE (MPa)	PRED. VALUE (MPa)	RESIDUAL	ACTUAL VALUE (MPa)	PRED. VALUE (MPa)	RESIDUAL
1	7.00	3.00	5.00	6.40	6.53	−0.13	73.70	73.67	0.027	0.11	0.11	0.00
2	7.00	4.00	5.00	7.22	7.27	−0.05	66.58	66.42	0.16	0.13	0.13	0.00
3	8.19	3.59	2.03	6.28	6.18	0.10	80.62	80.78	−0.16	0.12	0.12	0.00
4	7.00	3.00	5.00	6.53	6.53	3.03	73.53	73.67	−0.14	0.11	0.11	0.00
5	5.81	2.41	7.97	6.90	6.97	−0.07	57.01	57.10	− 0.0.09	0.14	0.14	0.00
6	7.00	3.00	5.00	6.72	6.53	0.19	73.70	73.67	0.03	0.11	0.11	0.00
7	7.00	3.00	5.00	6.49	6.53	−0.04	73.77	73.67	0.09	0.10	0.11	0.00
8	7.00	3.00	5.00	6.53	6.53	3.03	73.77	73.67	0.09	0.11	0.11	0.00
9	8.19	2.41	2.03	4.80	4.85	−0.05	81.99	82.10	−0.11	0.09	0.09	0.00
10	8.19	2.41	7.97	6.84	6.78	0.06	65.06	65.22	−0.16	0.12	0.12	0.00
11	5.81	2.41	2.03	4.17	4.09	0.08	59.29	59.46	−0.17	0.12	0.12	0.00
12	5.00	3.00	5.00	4.83	4.85	−0.02	52.87	52.70	0.17	0.15	0.15	0.00
13	7.00	3.00	5.00	6.50	6.53	−0.03	73.51	73.67	−0.16	0.10	0.11	−0.01
14	7.00	3.00	10.00	7.19	7.19	0.00	57.21	57.05	0.16	0.11	0.11	0.00
15	9.00	3.00	5.00	5.57	5.60	−0.03	77.31	77.13	0.18	0.12	0.12	0.00
16	7.00	3.00	0.00	4.27	4.32	−0.05	94.36	94.17	0.19	0.10	0.10	0.00
17	5.81	3.59	2.03	5.08	5.11	−0.03	59.77	59.86	−0.09	0.14	0.14	0.00
18	5.81	3.59	7.97	6.68	6.60	0.08	56.25	56.39	−0.14	0.12	0.12	0.00
19	8.19	3.59	7.97	6.68	6.72	−0.05	62.71	62.79	−0.08	0.12	0.12	0.00
20	7.00	2.00	5.00	6.46	6.46	1.15	68.32	68.13	0.19	0.10	0.11	−0.01

### Elongation at break (E_AB_)

Bio-composite films should, in general, be strong and flexible enough to bear different kinds of external stress and still fulfill the containment role without compromising their structural integrity. It is a crucial mechanical characteristic in exploiting polymers for packaging advancement. This characteristic is determined by calculating the proportion lengthening the barrier layer will accomplish before failing. It appears that CNC reduced the film’s elongation at break. For example, comparing the 0-weight per cent film of run 16 (94.36%) with 5-weight per cent and 10-weight per cent CNC reinforcement, respectively, to run 2 (66.58%) and run 14 (57.21%). According to the data, the EB dropped from 94.36 to 57.21%^16^. also noted a similar thing when they looked into how gelatin affected PVA/Gelatin mixtures. Higher EAB values are preferred for packaging applications, but they also need to be commensurate with the film’s resistance to the tensile stress needed to support the packing load.

### Thickness (TNS)

The gauge of the biodegradable film is a pivotal consideration since the structure affects the physical properties of the packaging Materials. The film thickness of nanocomposites is the single factor that affects their physical properties and is crucial for mechanical testing. Considering the 0-weight per cent film which is run 16 with 0.10 μm thickness as an example, compared to runs 2 and 14, which have 5 weight per cent and 10 weight per cent CNC reinforcement and 0.13 μm and 0.11 μm thickness, respectively. As demonstrated, the thickness increased from 0.1 μm to 0.13 μm. On the other hand, there was a Little 0.11 μm drop at the time the CNC was raised from 5 weight per cent up to 10 weight per cent which might have been caused by insufficient dispersion. A similar upward tendency was noted in another research^44^. Notably, the augmentation with CNC resulted in a modest rise in film thickness; nonetheless, there was no discernible variation in the gauge of the barrier layers. The gauge range found in this investigation is comparable to values found in other studies and is among the boundaries taken into consideration for food barrier layers^45,46^.

### Statistical analysis

After statistical examination of the experimental data using multiple regression analysis, as indicated in Table 2, the quadratic model exhibited the most effective power for evaluating TNS, E_AB_, and THS. A quadratic model was utilized for the purpose of examining the variance of the independent factors in relation to the outcome variables to evaluate the accuracy of the data. Moreover, the quadratic model achieved the highest value of the statistically modified R^2^, estimated R^2^, alongside R^2^.

**Table 2:** Summary of key model statistics.

Source	Std. Dev.			*R*-Squared			modified *R*-Squared			modified *R*-Squared			PRESS			Remark
	TNS	E_AB_	THS	TNS	E_AB_	THS	TNS	E_AB_	THS	TNS	E_AB_	THS	TNS	E_AB_	THS	
Linear	0.61	4.90	0.01	0.66	0.73	0.37	0.59	0.68	0.25	0.42	0.60	−0.05	9.96	573.9	4.67	
2FI	0.58	4.61	0.01	0.74	0.81	0.56	0.62	0.72	0.35	0.48	0.49	−0.06	8.97	732.6	4.69	
Quadratic	0.09	0.20	4.30	0.99	0.99	0.95	0.98	0.99	0.92	0.97	0.99	0.78	0.45	2.55	*9.52*	*Suggested*
Cubic	0.10	0.25	3.88	0.99	0.99	0.97	0.98	0.99	0.93	0.92	0.95	0.31	1.35	68.87	3.01	Aliased

TNS = Tensile strength, E_AB_= Elongation at break, THS = Thickness.

After that, a final model equation for predicting the responses for a specific amount of each element was created, as illustrated in equations (TNS), (EAB), and (THS). However, whereas the minus indicators suggest contrary impacts pertaining to the feedback, the beneficial indicators demonstrate the favorable outcomes of the components of the feedback. As a result, it was found that the tensile strength response coefficients for A (gelatin), B (CMC), C (CNC), AB (interplay amidst gelatin along with CMC), and B2 are beneficial. However, in comparison to their interplay with CNC led to unfavorable outcomes for the EAB, A (gelatin), B (CMC), and C (CNC) contributed positively to the elongation at break. It is evident that the CMC and CNC have the highest regression coefficients for tensile strength, but the CMC and gelatin coefficients are connected to elongation at break. Once more, it was noted that the thickening response coefficients for B (CMC), C (CNC), AB (gelatin and CMC interaction), AC (gelatin and CNC interaction), A2, and B2 are all positive. Thus, it can be concluded that whilst gelatin and CMC had the biggest effect on the elongation at break, CMC alongside CNC held the most repercussions on tensile strength together with thickness. This is accurate since the CNC can reinforce the nanocomposite by adding an interconnection framework to the matrix that raises the TS. However, molecular mobility is hindered by the networked structure, which reduces the matrix’s capacity to stretch under stress.

Where A, B, and C symbolize the unique identifiers of the independent variables.

### Statistical optimization of response surfaces

**Fig. 8:**
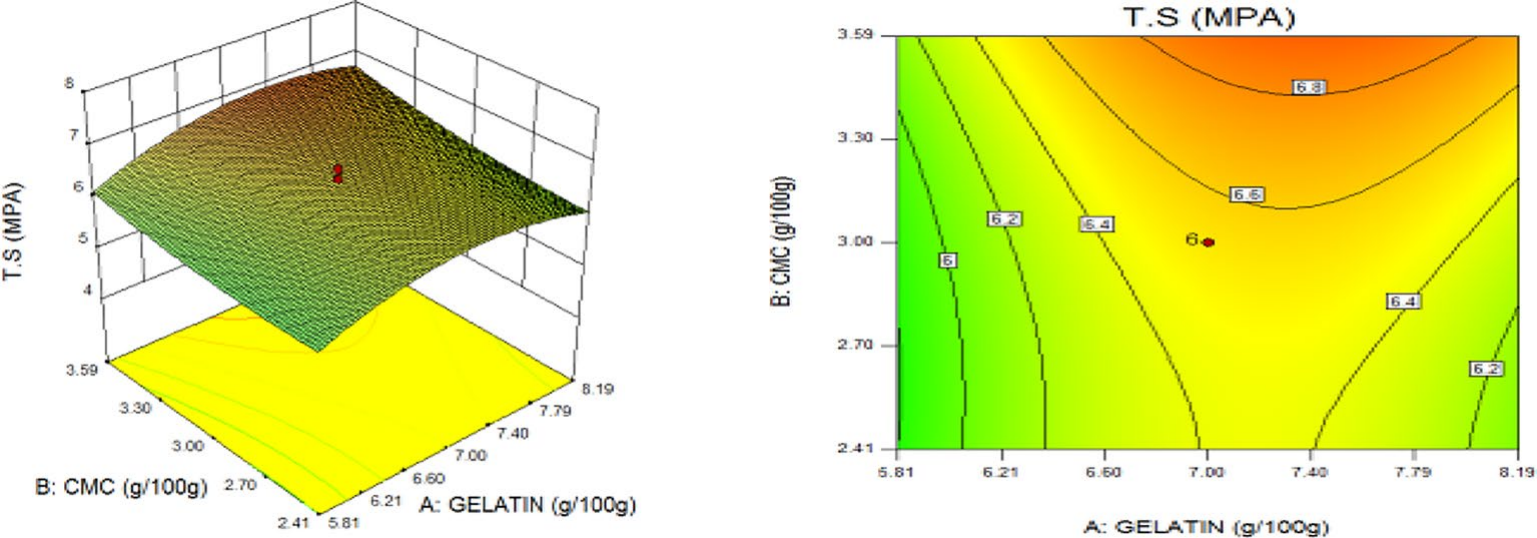
Gelatin and CMC influence on TNS.

**Fig. 9:**
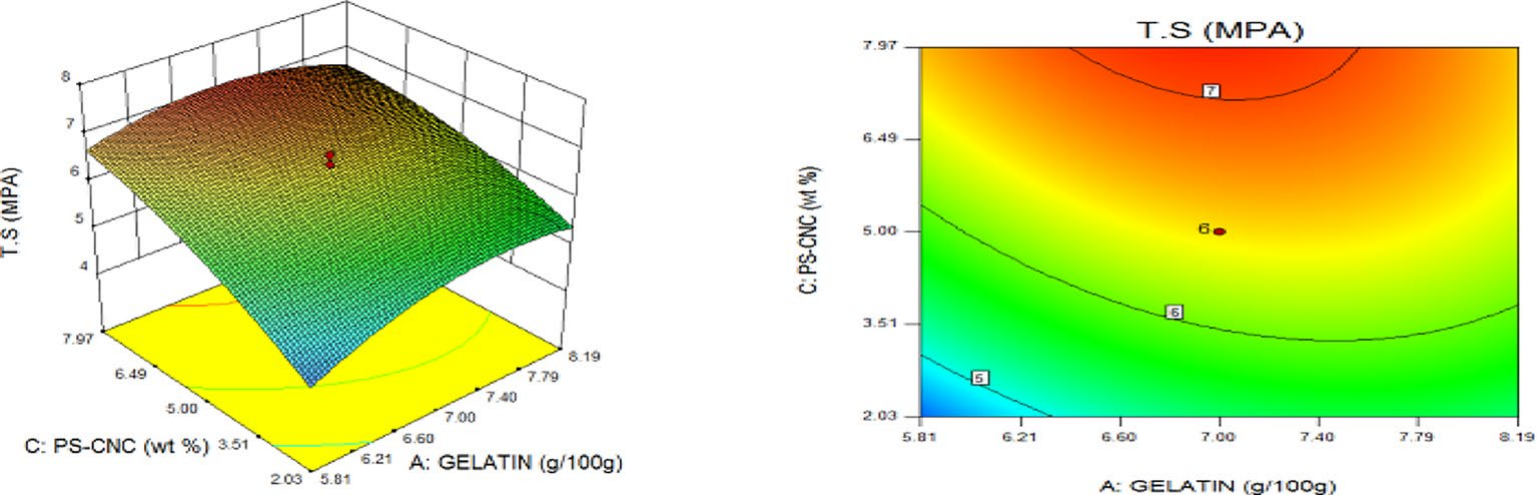
5-weight per cent CNC and Gelatin influence on TNS.

**Fig. 10:**
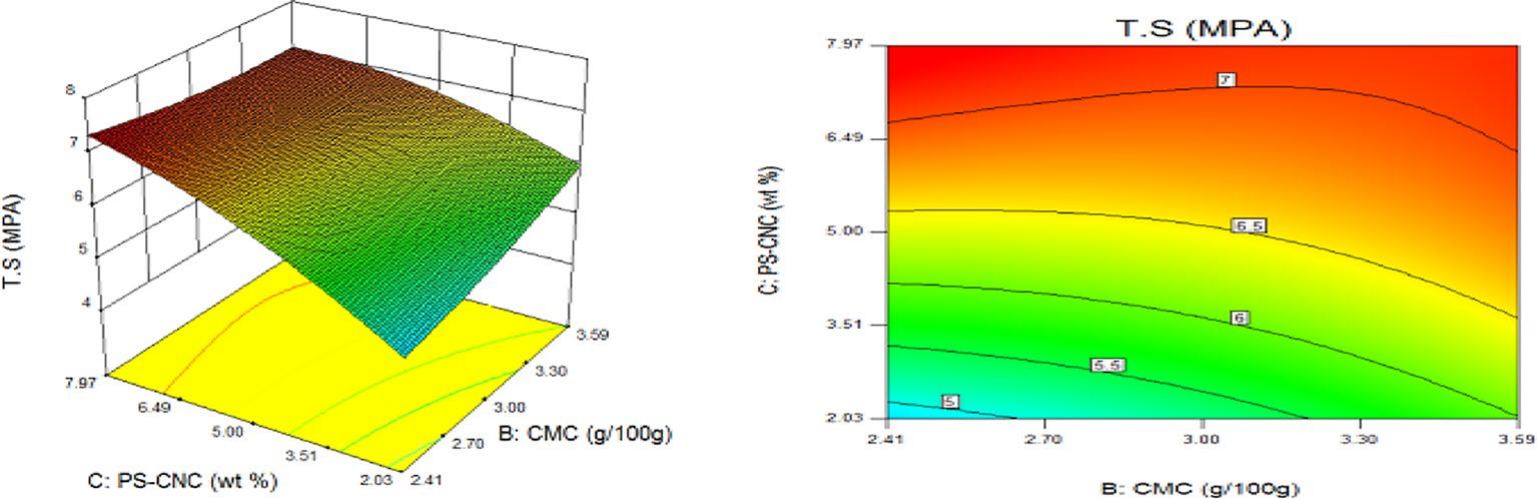
10-weight per cent CNC and CMC influence on TNS.

**Fig. 11:**
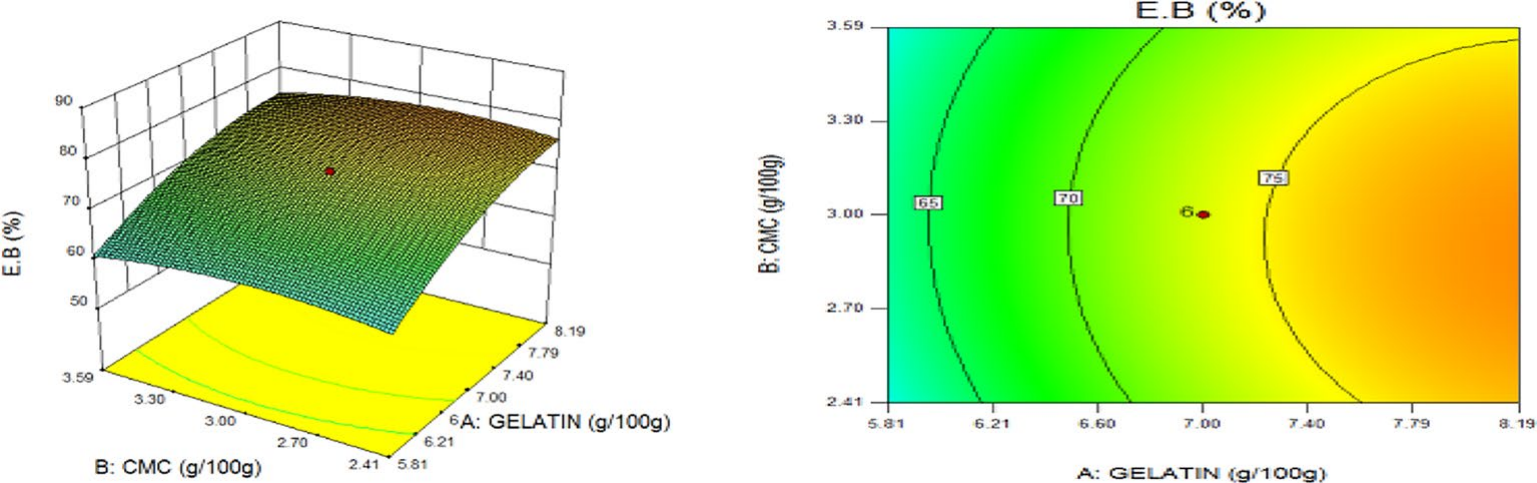
gelatin and CMC influence on E_AB_.

**Fig. 12:**
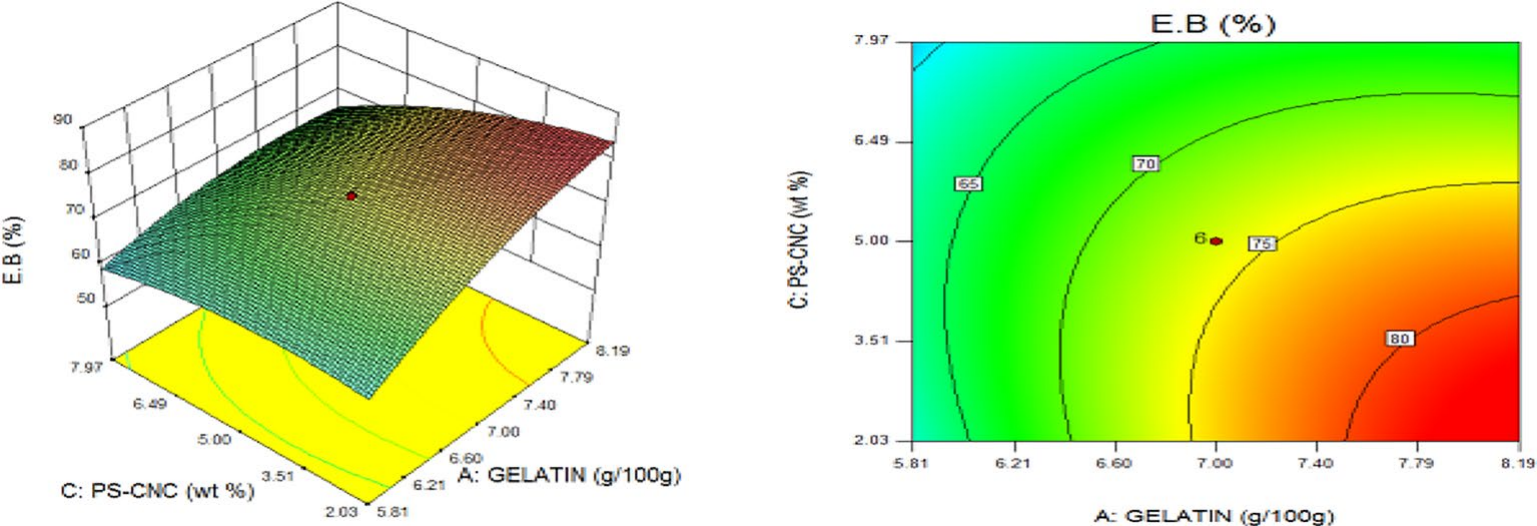
5-weight per cent CNC and gelatin influence on E_AB_.

**Fig. 13:**
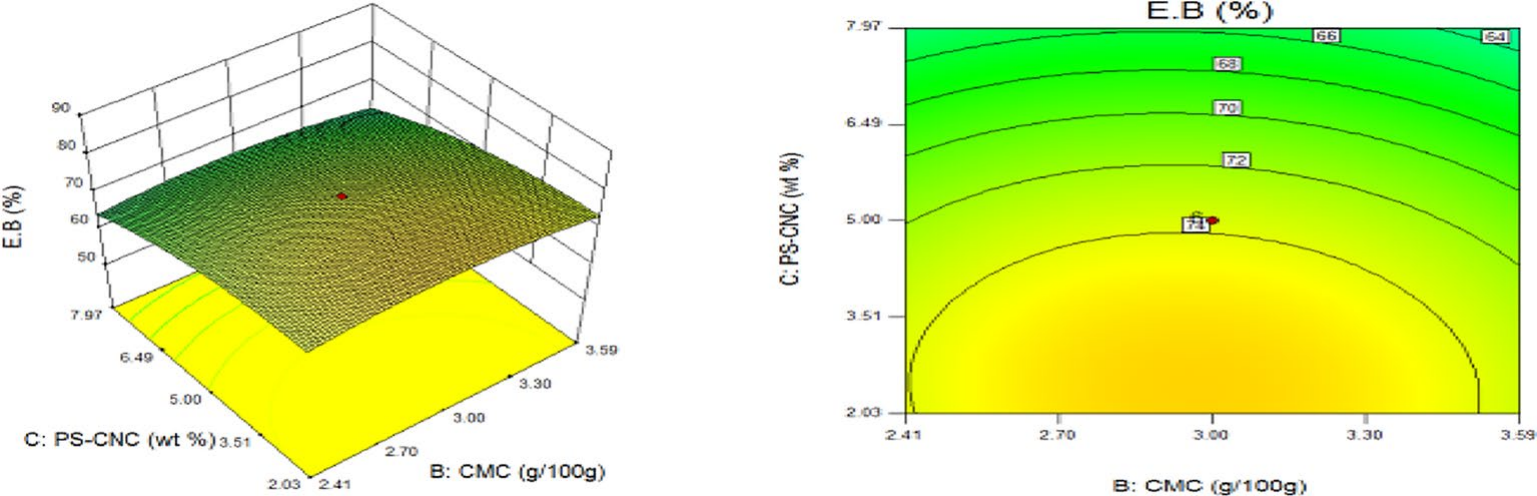
10-weight per cent CNC and CMC influence on E_AB_.

**Fig. 14:**
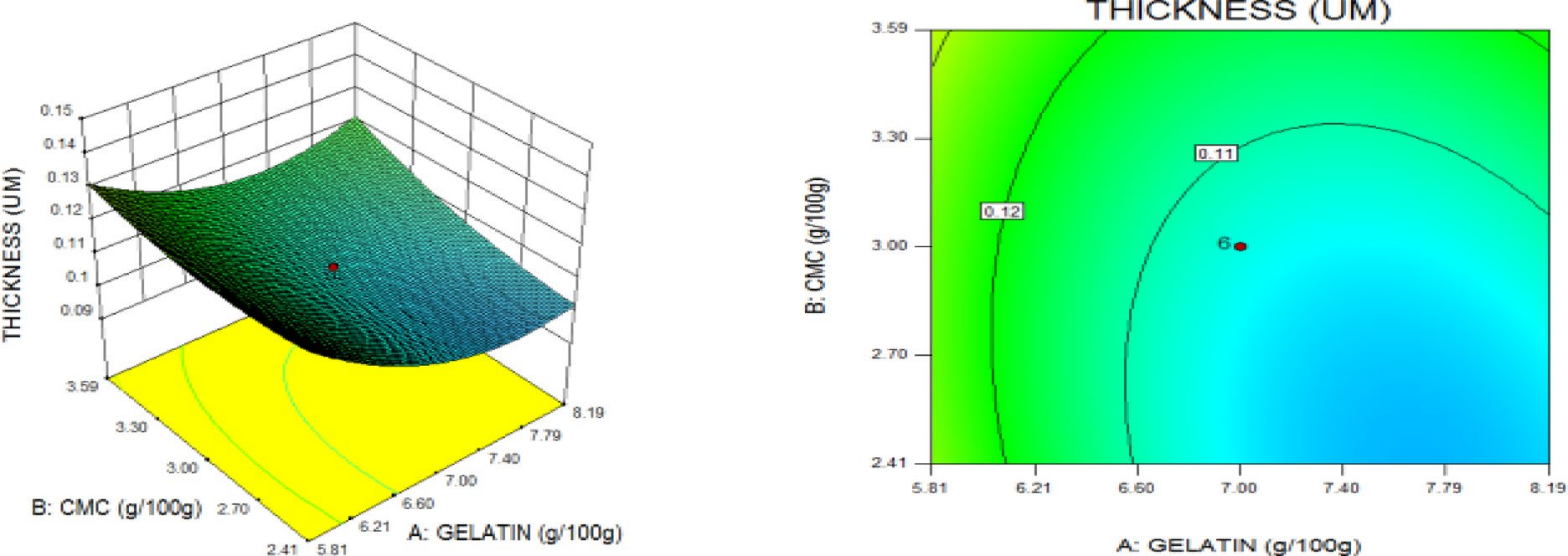
Gelatin and CMC influence on THS.

**Fig. 15:**
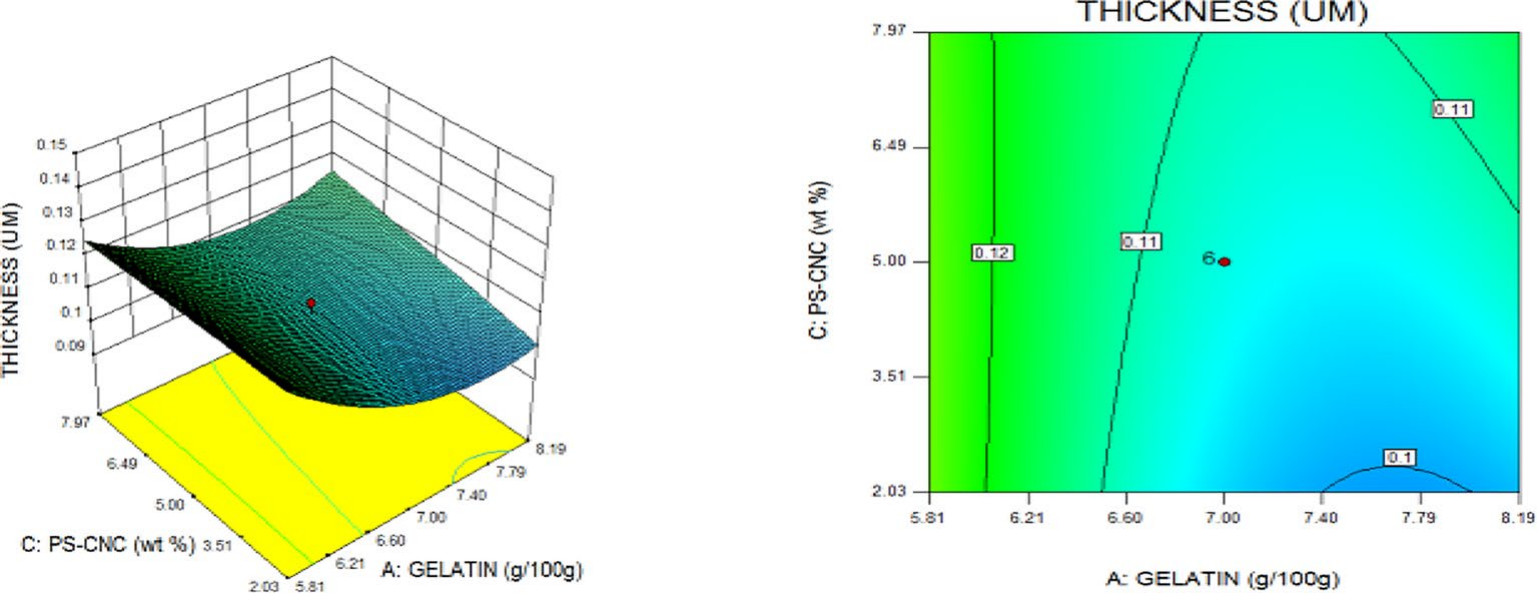
5-weight per cent CNC and gelatin influence on THS.

**Fig. 16:**
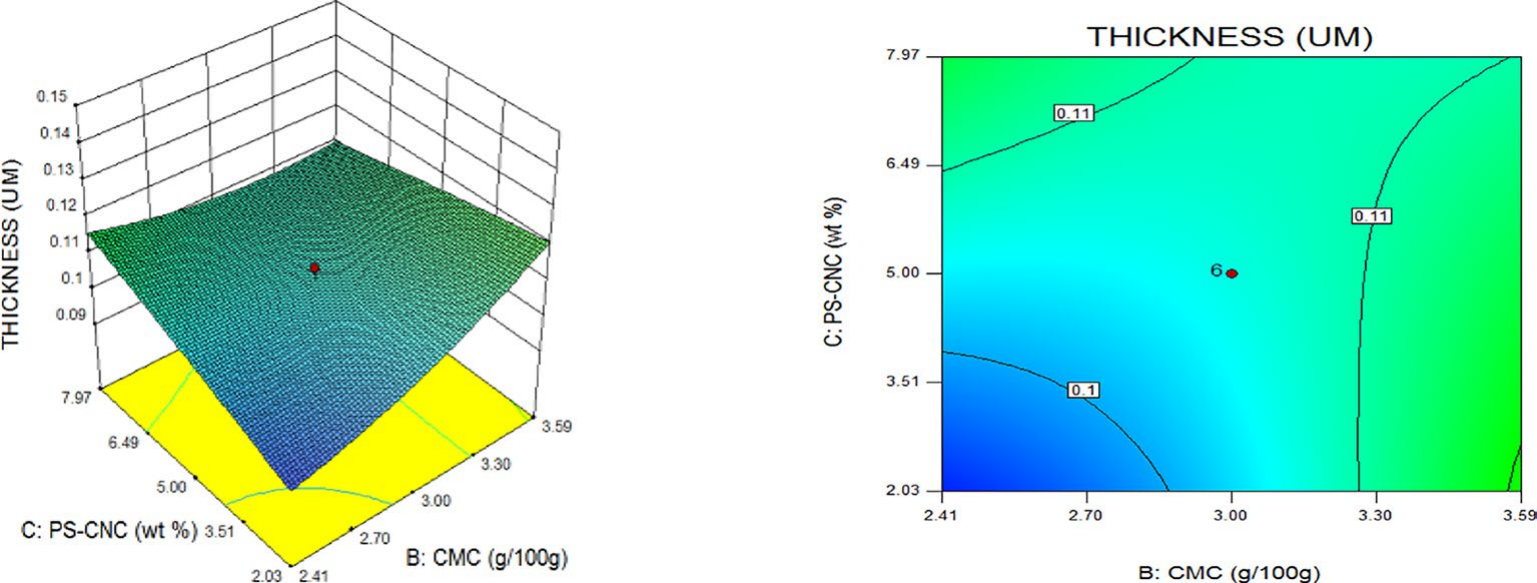
10-weight per cent CNC and CMC influence on THS.

The 3D and contour plots were created using the statistically significant factor that was found through statistical research. The numbers displayed represented the cumulative impacts of the three independent components (CMC, CNC alongside gelatin) with regards to TNS, THS alongside EAB. Two factors were examined in relation to the replies, with a constant third component. Higher concentrations of CMC and gelatin resulted in a commensurate incremental gain in tensile strength, as seen in Fig. 8. Nevertheless, an optimal result was seen when the amount of CMC was increased to its maximum, but the amount of gelatin could not reach its maximum input. This is further supported by Fig. 9, which studies the interaction between the CNC and gelatin while maintaining a constant CMC at its highest input. The primary contributors to the tensile strength are undoubtedly CMC and CNC, probably as a result of their molecular structural arrangement, whereas gelatin just served as a plasticizer to increase the nanocomposite’s ability to form films. The use of plasticizers has been shown to reduce TNS by modifying the polymer molecular arrangement by reducing intermolecular pressures [49]. This, in turn, diminishes film brittleness and enhances polymer adaptability. Figure 10 supports this by showing that the composite Materials at the nanoscale achieved an ideal TNS of approximately 6.5 MPa when the effects of CMC and CNC are synergistic whilst maintaining a constant gelatin content. The behavior was associated with the increasing augmentation of CMC alongside CNC supply. Whilst^48^ alongside^49^ confirmed that the combination of CMC and CNC increased the TNS of nanocomposites^50^, observed and noted the behavior of CMC in CMC/gelatin blends. In the meantime, 3D and contour plots of elongation at break made with statistically significant components from the CMC and gelatin ANOVA are displayed in Figs. 11, 12 and 13. This illustrated that CNC is necessary for optimal E_AB_ which was accomplished in the nanocomposite joined by a continual step-by-step addition of gelatin and CMC. This coincides with a previous observation^50^, which discovered that biodegradable films composed of a composite of starch and PVA had high TNS of 16.22 MPa and E_AB_ of 116.12%. However, the ideal E_AB_ disappeared to the peripheral when CNC was gradually added in Figs. 12 (5%) and 13 (10%), leaving only individual contributions to be felt. This lends further credence to the idea that gelatin was a key factor in terms of the nanocomposite’s plasticization and film-forming capabilities. In addition, it is critical to remember that TNS is increased and E_AB_ is decreased because CNC networks the nanocomposite’s structure. Since greater elongation at break is mostly caused by molecular mobility, networked structures in the case of E_AB_ impede it. Meanwhile, Figs. 14, 15 and 16 show the construction of 3D and thickness contour plots using statistically important components based on the analysis of variance of CMC and gelatin.

### Optimizing experimental conditions

Table 3, illustrates how the experimental design was used to determine the ideal concentrations of values CMC, gelatin, and CNC, resulting in optimal values for TNS, E_AB_, and THS. The best results were achieved at a desired level of 72%, with CMC of 4 weight per cent, gelatin of 7 weight per cent, and CNC of 5 weight per cent. The corresponding predicted values for TNS, E_AB_, and alongside thickness, were 6.47 MPa, 78.92%, and 0.12 μm, respectively. Confirmation experiments were run, and the results were compared to the expected values in order to test the models. The experimental values differed from predicted values by a percentage of error less than 10%, a good agreement could be shown from the comparison.

**Table 3:** Verification test results.

PS	Predicted values	Experimental values	% Error
TS (MPa)	6.47	6.63	4.57
E_B_ (%)	78.92	79.56	0.80
TK (um)	0.12	0.13	7.7

Tensile strength = TNS, Elongation at break = E_AB_, Thickness = THS.

### Total dissolved solid

**Fig. 17:**
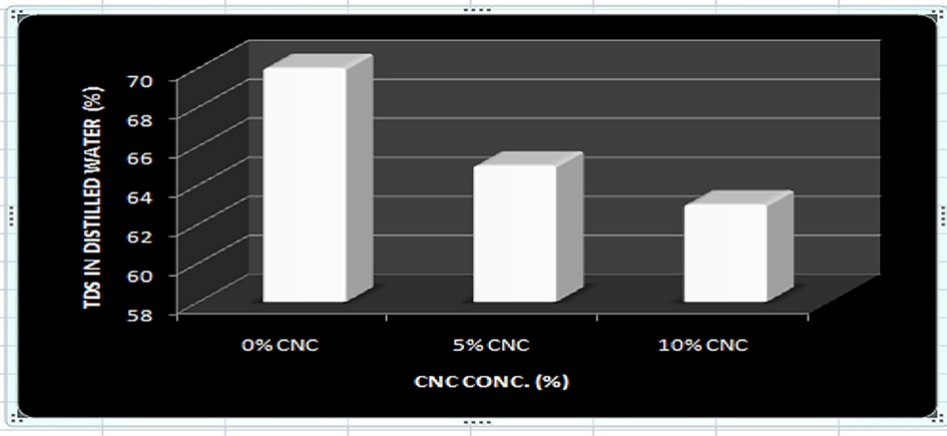
Total dissolved solid in distilled water.

The complete dissolved material from among reinforced and strengthened CMC/gelatin nanomaterial composites shown in Fig. 17. A decrease in the effectiveness of weight downturn was seen upon the incorporation of CNCs into the CMC-Gelatin Matrix. TDS in the 0-weight per cent films were 70%, but TDS in the 5-weight per cent and 10-weight per cent CNC reinforced films were 65% and 63%, respectively. The formation of a robust strong hydrogen connection between the crystalline nanocellulose and matrix is responsible for the decrease in solubility. By creating a three-dimensional network, the water sensitivity reduced and limited the polymeric matrix’s ability to dissolve in water^51^.

**Fig. 18:**
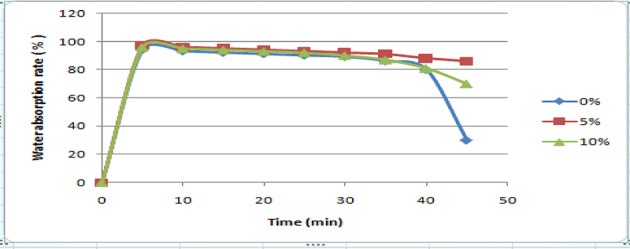
Water absorption rates of nanocomposite films on distilled water.

An essential characteristic for assessing polymer films’ long-term performance is their liquid assimilation capacity. Figure 18 displayed the liquid assimilation results from among CMC/gelatin film that was 0-weight per cent and the CMC/gelatin film that was reinforced with 5 weight per cent and 10 weight per cent CNC. Instead of showing equilibrium absorption, the 0-weight per cent film’s absorption progressively declined over time. Before deteriorating, the 5-weight per cent and 10-weight per cent plantain stem CNC reinforced film kept equilibrium water absorption for 20 min. Nevertheless, the reinforced films retained their equilibrium water content throughout after 40 min of absorption and degradation, while the unreinforced film kept going downhill. Furthermore, beyond 30 min, the unstrengthened film assimilated roughly 89% of its initial Mass, compared to absorption rates of 62% and 60% for the 5-weight per cent and 10-weight per cent CNC reinforced films, respectively. Consequently, the addition of CNC to the mixture reduced its water absorption due to the highly ordered three-dimensional assemblies that were created via hydrogen bridges connecting the molecules of CNC alongside the polymeric matrix^51^.

### Moisture uptake

**Fig. 19:**
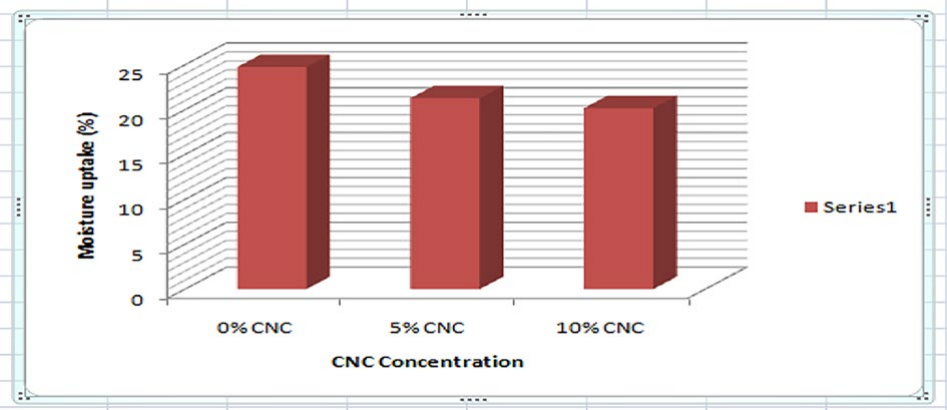
Moisture uptake of the nanocomposite films.

When it comes to packaging, especially for food preservation, a film’s capacity to withstand moisture is essential. However, one of the shortcomings of CMC-Gelatin films is their ineffective moisture resistance. Figure 19 illustrates how the inclusion of CNC brought about a decrement in the films’ moisture absorption. The moisture uptake of the 0-weight per cent CMC-Gelatin film was 24.75%; however, when 5-weight per cent and 10-weight per cent of CNC was added, the moisture uptake decreased to 20.17% and 21.28 weight per cent respectively. According to reports, when 10% CNC is added to starch, moisture uptakes drop from 13.8%^35^. Food packaging has a corresponding increasing effect, as food production and food security, this is critical in national development^52^, efficient food packaging food material is therefore critical for national development.

### Moisture uptake

**Fig. 20:**
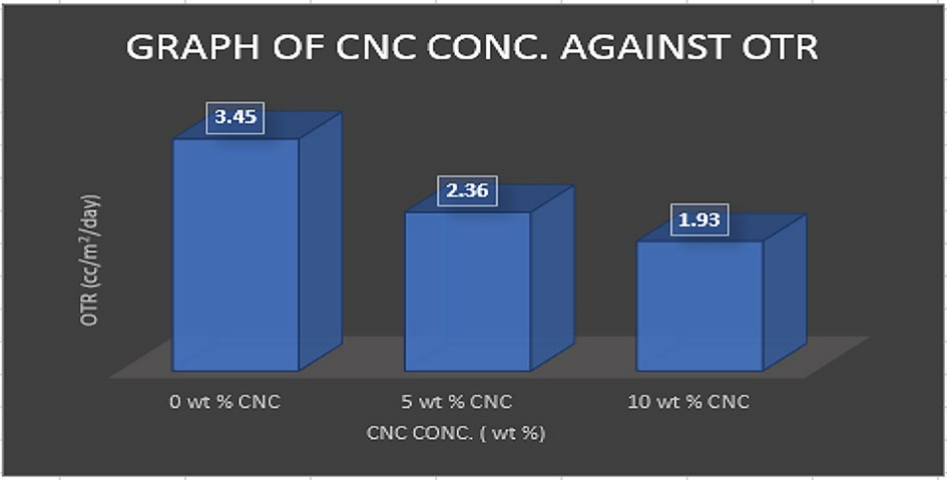
Oxygen transmission rate of the nanocomposite.

Due to deterioration, which alters food’s color, odor, and flavor while also degrading nutrients, OTR is an essential metric for assessing the efficacy of foods packaging substances^38^. For packaging-related uses, OTR levels less than twenty cc/m^2^/day were suggested. Therefore, CMC and gelatin-reinforced CNCs made from plantain stems can provide a suitable O2 protection that may help to increase the nutritional value as well as preservation time. Figure 20 shows the results of an investigation into the OTR of 0wt% CMC-Gelatin films, 5wt%, as well as 10wt% CNC reinforced CMC-Gelatin films.

OTR for 0wt% CMC-Gelatin films were 3.45 cc/m^2^/day, according to the investigation’s findings, however it decreased to 2.36 cc/m^2^/day and 1.93 cc/m^2^/day for 5wt% and 10wt% CNC reinforced films, accordingly. A sign of how the OTR of the resulting nanocomposite films improved when CNC was added to the CMC-Gelatin mixture. Remarkably, the OTR found in the present investigation is somewhat lower compared to the OTRs of 3.63, 4.26, and 5.23 cc/m^2^/day observed for the CNCs/PLA/MnO_2_-NPs mixture^53^. The reduction in OTR is ascribed to the impenetrable CNC that was well disseminated in the mixture medium to generate a convoluted channel and to increase the effective diffusion path length for OTR^55^. Interestingly, film’s with 5 and 10 wt% have lesser OTRs than the 0 wt% film.

## Conclusion

Utilizing plantain stem fiber, cellulose nanocrystals with a diameter spanning from 20 nanometers to 50 nanometers and 82% crystallinity were produced. These nanocrystals were subsequently strengthened and used to construct nanomaterial composite films with gelatin alongside CMC providing the framework for potential product packaging solutions. Using the central composite design, the impacts of gelatin, CMC, alongside CNC on the mechanical parameters (TNS, E_AB_, and thickness) were examined. The investigation’s findings demonstrated that the created gelatin, carboxymethyl cellulose, and CNC films had slender shapes possessing lengths between 81 and 286 nanometers and diameter between 8 and 21 nanometers, with a high crystallinity index of 0.82 and an elongated aspect ratio of 17 when observed under scanning electron microscopy. When 5-weight per cent alongside 10-weight per cent portions of CNC were added, there was a consistent dispersal of CNC throughout the framework to produce uniformly thin films, showing that the CNC and CMC/gelatin were well Matched. With the incorporation of CNC, the thickness of the nanomaterial composite films grew stemming from 0.1 μm to 0.11 μm, and their TNS elevated from 4.27 MPa to 7.22 MPa. Additionally, their E_AB_ diminished from 94.36 to 57.21%. The nanocomposite films TDS dropped from 70 to 63% as well. Although CNC is hydrophilic, its enhanced water barrier characteristic following addition can be attributed to its ability to construct a compact, 3-D structure in combination with the matrix that keeps moisture molecules out. In conclusion, the findings highlight the potential of CNC from the stem of plantain-reinforced gelatin/CMC films as a biodegradable packaging material, particularly for food that is being transported.

### Limitations of the study

One of the limitations of this study includes the use of acid hydrolysis rather than enzymatic hydrolysis to produce CNC. The amorphous portion of cellulose was eliminated via chemical hydrolysis procedures using sulfuric acid hydrolysis in order to produce CNC. This is related to the high cost of generating CNCs using enzymatic hydrolysis, which is regarded to be a more sustainable alternative. Enzymatic hydrolysis offers more benefit by using less water for washing, produces no hazardous chemical residues, works under mild reaction settings, and is more environmentally friendly. This study was limited to three mechanical properties viz.: tensile strength, elongation at break and thickness. Other mechanical properties such as flexural strength, hardness, puncture and tear resistance, creep behaviour, were not considered. The optimization of more factors will definitely have implication on the outcome of the mechanical property of the bio-package, this is a limitation on this study. In addition, effort to improve the mechanical properties of the bio-packaging material was limited to reinforcing fibers (CNC) without considering the impact of additives or other materials. The blending of the CNC to improve the properties of the bio-package was limited to zero (0), five (5) and ten (10) percent. More blending ratios may present outcome that is not fully captured in this study. This study failed in scope to consider other barrier properties such as gas/aroma barrier, light and Oxygen barrier. This study is also limited in providing information on the economics of commercialization of the bio-package product.

## Data Availability

All data generated or analysed during this study are included in this published article.
